# Tumour-induced osteomalacia: a literature review and a case report

**DOI:** 10.1186/s12957-015-0763-7

**Published:** 2016-01-08

**Authors:** Jolanta Dadoniene, Marius Miglinas, Dalia Miltiniene, Donatas Vajauskas, Dmitrij Seinin, Petras Butenas, Tomas Kacergius

**Affiliations:** 1 Faculty of Medicine, Vilnius University, Ciurlionio 21, Vilnius, Lithuania; 2 Vilnius University Rheumatology Centre, Santariskiu 2, Vilnius, Lithuania; 3 State Research Institute for Innovative Medicine, Zygimantu 9, Vilnius, Lithuania; 4 Vilnius University Centre of Nephrology, Santariskiu 2, Vilnius, Lithuania; 5 Vilnius University Radiology and Nuclear Medicine Centre, Santariskiu 2, Vilnius, Lithuania; 6 National Centre of Pathology, Baublio 5, Vilnius, Lithuania; 7 Vilnius University Hospital Santariskiu Klinikos Orthopaedic Traumatology Department, Santariskiu 2, Vilnius, Lithuania

**Keywords:** Tumour-induced osteomalacia, Hypophosphataemia, Fibroblast growth factor 23

## Abstract

Tumour-induced osteomalacia (TIO) is a rare paraneoplastic syndrome characterised by severe hypophosphataemia and osteomalacia, with renal phosphate wasting that occurs in association with tumour. The epidemiology likewise aetiology is not known. The clinical presentation of TIO includes bone fractures, bone and muscular pains, and sometimes height and weight loss. TIO may be associated with mesenchymal tumours which may be benign or malignant in rare cases. Mesenchymal tumour itself may be related to fibroblast growth factor 23 (FGF23), which is responsible for hypophosphataemia and phosphaturia occurring in this paraneoplastic syndrome. Hypophosphataemia, phosphaturia and elevated alkaline phosphatase are the main laboratory readings that may lead to more precise investigations and better diagnosis. Finding the tumour can be a major diagnostic challenge and may involve total body magnetic resonance imaging, computed tomography and scintigraphy using radiolabelled somatostatin analogue. The treatment of choice for TIO is resection of a tumour with a wide margin to insure complete tumour removal, as recurrences of these tumours have been reported. We provide here an overview on the current available TIO case reports and review the best practices that may lead to earlier recognition of TIO and the subsequent treatment thereof, even though biochemical background and the long-term prognosis of the disease are not well understood. This review also includes a 4-year-long history of a patient that featured muscular pains, weakness and multiple stress fractures localised in the hips and vertebra with subsequent recovery after tumour resection. Because the occurrence of such a condition is rare, it may take years to correctly diagnose the disease, as is reported in this case report.

## Background

Tumour-induced osteomalacia (TIO) is a rare paraneoplastic syndrome clinical presentation of which includes bone fractures, bone and muscular pains, and sometimes loss of height and even weight. Weight loss is unusual, but sometimes observed, and could be explained by general debilitated state of the patient with consequent poor nutrient intake and loss of muscle mass [1, 2]. Most of them are believed to be benign, although cases judged to be histopathologically malignant have been also described [3–6]. Mesenchymal tumour itself may be related to fibroblast growth factor 23 (FGF23), which is responsible for hypophosphataemia and phosphaturia occurring in this paraneoplastic syndrome. Hypophosphataemia, phosphaturia and elevated alkaline phosphatase are the main laboratory readings that may lead to more precise investigations and better diagnosis. Although several hundreds of cases have been reported since 1947 when McCance et al. described the first TIO case [7], majority of clinicians, radiologists and pathologists are not aware of this rare disease with only several cases described in rheumatology practice [5, 6, 8–14]. We provide an overview of the current literature available on TIO case reports and review the best practice for the diagnosis and treatment. We also present here a 4-year-long case study of a patient who was eventually diagnosed with TIO. We report the initial presentation of the disease and the steps leading to TIO diagnosis.

## Review

### Disease name and definition

TIO is characterised by severe hypophosphataemia and osteomalacia, with renal phosphate wasting that occurs in association with tumour. TIO also is called oncogenic hypophosphatemic osteomalacia (M83.8) and appears in the portal for rare diseases and orphan drugs under that name (ORPHA352540). It was first described in 1947 by Robert McCance, who reported a patient with pain, weakness, gait abnormalities, and low phosphorus levels. The patient was treated with high doses of vitamin D, but the symptoms did not completely resolve until a tumour in femur bone was removed [7].

The first person to clearly recognise that the disease was the result of a “rachitogenic” substance was Andrea Prader. In 1959, he described an 11 1/2-year-old girl who developed severe rickets over the course of a year. Evaluation showed decreased tubular phosphate reabsorption but otherwise normal kidney function. A tumour, classified as a giant cell granuloma, was identified in a rib and removed resulting in healing of rickets [15].

### Epidemiology

The prevalence of the disease is not known. Since the association between phosphate reabsorption and tumour was first made, more than 300 cases of TIO have been reported in the literature [16].

### Pathogenesis

The first evidence of a circulating factor that could cause phosphate wasting in phosphaturic disorders such as TIO was demonstrated in a set of experiments in mice by Meyer et al. and Nesbitt et al. [17, 18]. The first evidence to support this concept in humans was the experiment by Miyauchi et al. in which a tumour removed from a patient and transplanted into nude mice caused hypophosphatemia [19].

FGF23 was first identified as the phosphaturic substance when mutations in FGF23 were linked to autosomal-dominant hypophosphatemic rickets (ADHR) [20, 21]. Soon after that, elevations in serum FGF23 were found in TIO and it was shown that FGF23 binds to target proximal tubule cells via FGF receptor and inhibits renal phosphate reabsorption [21]. The primary transport protein responsible for phosphate reabsorption in the kidney is the type II sodium-phosphate co-transporters (NaPi-IIa and NaPi-IIc) localised in the proximal tubule. High circulating FGF-23 levels reduce the expression of the co-transporters, leading to renal phosphate wasting [16, 22–24]. FGF23 is also a regulatory hormone for 1.25-vitamin D and leads to a decreased concentration of the vitamin in blood [25].

Excessive FGF23 action causes several hypophosphatemic diseases, including TIO, X-linked hypophosphatemic rickets (XLHR), autosomal dominant and recessive hypophosphatemic rickets (ADHR and ARHR) [26–28]. Under physiological conditions, FGF23 is secreted predominantly by bone and undergoes degradation by proteolytic enzymes [27]. By contrast, tumours secrete FGF23 to concentrations that are several hundred-fold higher than normal levels, leading to dysregulation of FGF23 degradation pathway [29].

### Histopathology

The tumours associated with TIO are usually small and mesenchymal in origin [30]. The prototypical phosphaturic mesenchymal tumour (mixed connective tissue variant) contains neoplastic cells that are spindled to stellate in shape, normochromatic with small nuclei and indistinct nucleoli. The nuclear grade is low, and mitotic activity is usually absent or very low. The cells are typically embedded within a myxoid or myxo-chondroid matrix with “grungy” calcification that can resemble chondroid or osteoid. Numerous osteoclast-like giant cells are a frequent finding, and mature fat and even lamellar bone may also be seen. A prominent feature of these tumours is an elaborate intrinsic microvasculature with an admixture of vessel size and vascular pattern [3]. The most common diagnosis for these tumours has been hemangiopericytoma, but it has also included hemangioma, sarcomas, ossifying fibromas, granulomas, giant cell tumours and osteoblastomas.

Weidner in 1991 was the first to propose a classification system based on the histological findings of 16 cases of TIO and designated the tumours as phosphaturic mesenchymal tumours [31]. TIO tumours can be further subdivided into four categories: mixed connective tissue variant (phosphaturic mesenchymal tumour mixed connective tissue variant—PMTMCT), osteoblastoma-like variant, non-ossifying fibroma-like variant, and ossifying fibroma-like variant. The PMTMCT group is diagnosed in 70–80 % of TIO cases and comprises neoplasias containing primitive stromal cells, prominent vessel, and osteoclast-like giant cells. PMTMCT tumours usually occur in bones or soft tissues and are typically benign in behaviour, but malignant variants have already been described. Malignant examples show frankly sarcomatous features, such as a high nuclear grade, high cellularity, elevated mitotic activity and necrosis [3, 6, 22, 32]. The remaining three groups tend to occur in bones and were also typically benign in behaviour.

When testing antigen expression, FGF23 is positive in about 70 % of all the cases studied, and the proliferating cells within the tumour are usually the source of FGF23 [3]. Somatostatin receptors have also been found to be present in many TIO tumours [33–35].

While typically benign, malignant presentation and metastases can occur [4, 5, 36]. While metastases are rare, infiltration of surrounding connective tissue is typically present, which has significant implications for surgical management and emphasises importance for wide surgical margins to avoid persistence or reoccurrence.

Regardless of tumour morphology, the hallmark of the diagnosis is the association of the tumour with the clinical syndrome of TIO, which includes an elevation in plasma FGF23 and its disappearance after tumour resection.

### Clinical evaluation and diagnosis confirmation

Quoting Jiang et al. who reviewed 308 tumour-induced osteomalacia cases reported in English literature between 1987 and 2011, about 46 % of reported cases of TIO have occurred in females and 56 % in males, with a mean age of 45.3 years when definitive diagnosis was made [37]. Within the reported cases TIO, tumours originate in bones (40 %) and soft tissues (55 %). Most tumours are reported to occur in the thigh and femur (22.7 %), craniofacial region (20.7 %), ankle and foot (8.8 %), pelvis (8.2 %), tibia and fibula (6.5 %) and arms (6.5 %). The less common locations are the vertebra, knee, hand, chest, abdomen, groin, perineum and gluteal region [3, 37, 38]. Some tumours can even be located in organs such as the liver, tongue, thyroid and lungs [1, 3, 4, 39]. A few patients (2 %) are reported as having tumours in more than one site, sometimes representing metastases [37].

Patients with TIO often present with many years of symptoms before they are diagnosed. The symptoms usually are nonspecific and often progressive. Common complaints are bone pain, muscle weakness, reduced height, and multiple fractures, primarily in the ribs, vertebral bodies, and femoral neck [22, 37]. The patients are often misdiagnosed with a variety of musculoskeletal, rheumatologic diseases and sometimes even psychiatric disorders [16, 40]. Hypophosphatemia caused by impaired renal phosphate reabsorption is the biochemical hallmark of the disease. Additional laboratory tests can be helpful in making the diagnosis of TIO. The typical biochemical pattern of TIO includes normal or low levels of 1,25-dihydroxyvitamin D, elevated levels of alkaline phosphatase (reflecting osteoblast hyperactivity and active bone remodelling) and normal circulating levels of calcium and parathormone (PTH). In some cases, PTH can be particularly high reflecting secondary hyperparathyroidism, which is a normal response to low 1,25-vitamin D caused by elevated FGF23 [22, 23, 41].

Differential diagnosis should always include renal Fanconi’s syndrome—a disorder of the proximal renal tubules, leading to impaired phosphate reabsorption and hypophosphatemia. This syndrome may be genetic in origin or it may occur as a complication of myeloma, amyloidosis, or Sjogren’s syndrome. It can also occur with certain medications or heavy metal poisoning. The diagnosis can be confirmed by normal levels of FGF23 along with the presence of glycosuria, hypokalaemia, and metabolic acidosis [2].

Once the diagnosis of an FGF23-dependent, phosphate wasting disorder is made, a thorough history can aid in excluding the genetic causes, such as XLHR, ADHR and ARHR. Genetic testing can also be done.

Having narrowed the diagnosis to TIO, a careful physical examination should be performed, as the tumours that cause TIO can sometimes be found in the subcutaneous tissue [42]. As tumours can arise in bone or soft tissue, occur from head to toe and are typically very small in size and slow-growing, locating these tumours is often quite challenging. As a result, the time from osteomalacia to identifying the associated tumour averages a period of 5 years [43].

Finding the tumours can be a major diagnostic challenge and may involve total body magnetic resonance imaging (MRI); as tumours can occur anywhere in the body, it is important to scan the whole body, including extremities, which is often excluded in routine nuclear medicine imaging [44], computed tomography (CT), scintigraphy using radiolabelled somatostatin analogue (such as 99mTc-Tektrotyd) and positron emission tomography, with computed tomography: fluorodeoxyglucose (18F-FDG) PET/CT and galium (68Ga) DOTATATE PET/CT and selective venous sampling for FGF23 [44–51].

A stepwise approach is advocated, first performing functional tests. Tumours associated with osteomalacia variably express five somatostatin receptors (SSTR1-5), allowing SSTR-based functional imaging by somatostatin analogue scintigraphy or positron emission tomography [33, 46, 52, 53]. Octreotide is somatostatin analogue used in treatment of some neuroendocrine tumours and acromegaly. It is possible to radiolabel the somatostatin analogue in an attempt to detect tumours that express somatostatin receptors [38, 47, 52]. Octreotide scanning is commonly performed with ^111^In-labelled pentatreotide. Octreotide scintigraphy is successfully used to locate tumours in up to 95 % of patients with TIO [37]. It may even be possible to use this technology therapeutically in radioimmunoguided surgery or labelling of octreotide with a beta-emitting radionuclide [47]. Despite this success, there are several limitations of this imaging technology. Inflammatory reactions or a fracture will be associated with a false-positive scan. Somatostatin analogues scintigraphy is also limited by planar two-dimensional imaging and relatively poor spatial resolution, which is particularly problematic given that TIO tumours are often very small. Single-photon emission tomography (SPECT) or hybrid SPECT/CT enables three-dimensional imaging and better tumour contrast but is time-consuming and therefore limited to areas of suspected abnormality rather than a whole body survey [35]. Somatostatin analogue positron emission tomography can dramatically improve the spatial resolution and lesion detectability [54].

Anatomic imaging (radiography, CT and MRI) should be performed to confirm the location of the tumour after suspicious lesions had been identified by functional imaging [13]. High-resolution magnetic resonance imaging of the whole body is the currently proposed method of choice to confirm the location of the tumour [41].

Despite all of the advances in imaging that are available today, tumour localization may not be successful. If this is the case, imaging studies should be repeated every 1–2 years.

### Treatment

The treatment of choice for TIO is resection of a tumour with a wide margin to insure complete tumour removal, as recurrences of these tumours have been reported [4, 9, 36]. Tumour removal is always curative, and following complete resection of the tumour, the recovery and improvement of the patients is relatively quick, FGF23 disappears rapidly from the circulation, and serum phosphate returns to normal by day 5 post operation [55]. Most patients feel better within days to weeks of tumour removal. Bone healing starts immediately, but depending on the severity of the disease, it may take up to a year for a more significant clinical improvement to be seen.

In case of incompletely resected tumours, subsequent radiotherapy can be used to avoid recurrence or metastasis (41). Late recurrence due to metastatic disease is rare but possible. This probably occurs in less than 5 % of the patients with TIO [3, 36]. Lung is a common site for metastasis. The course after metastasis is quite variable, and survival of up to 30 years has been reported [56]. There is no chemotherapeutic regimen with any demonstrated efficacy in treating metastatic TIO. Radiofrequency ablation (RFA) has been reported as a possible treatment modality [14].

When the tumour cannot be localised or is not surgically resectable, medical therapy with phosphate supplementation and calcitriol or alfacalcidol is used. Treatment with octreotide is an alternative form of medical therapy that may be considered. In one case report, phosphate wasting and hypophosphatemia were corrected with octreotide therapy; this effect was presumably mediated by somatostatin receptor expression by the tumour, which was demonstrated by octreotide scintigraphy [43]. However, not all patients respond to octreotide [33, 57]. Treatment with the calcium-sensing receptor agonist, cinacalcet, could also be effective for the treatment of TIO patients by inducing hypoparathyroidism and thus increasing renal phosphate reabsorption [58].

Anti-FGF23 antibody is being studied as a novel therapy for FGF23-related hypophosphatemic diseases [59]. Results of phase I study of single injection of humanized anti-FGF23 antibody for adult patients with XLHR were recently published. This antibody therapy may be useful for patients with TIO [60, 61].

### Prognosis

The prognosis depends on detecting the tumour and possibility to remove it widely. The tumours when detected are typically benign in most of the cases. The symptoms disappear and the healing of the bones begin after total removal of the tumour. Nevertheless, the follow-up should be continued because the delayed metastasis can occur as it was described in few cases.

## Case report

A 48-year-old man was first seen by a rheumatologist in the beginning of 2011 because of progressing weakness, 10 kg weight loss during the recent year, diminishing height and muscular and bone pain in arms, lower back and chest which severely limited his movements. The patient felt unhealthy since 2009; however, his life history had no recorded health-related problems except for rib fracture during a car accident in his childhood and many years of smoking. His family history revealed that his father died of pancreatic cancer, his brother died of oncohematology condition and his mother lives with oncohematological disease. At the time of investigation, he was 180 cm tall (being 184 cm when healthy) and weighted 73 kg (BMI = 22.5 kg/m^2^). Physical examination revealed scoliosis and kyphosis of the spine, overall weakness, weakness in muscles and pain in both arms when palpating. The laboratory investigations showed no inflammation, and the only abnormality in serological readings was elevated alkaline phosphatase—248 U/l (normal values, 40–150) due to its bone-specific fraction comprising 80.5 % (Table 1). The radiology of the spine revealed compressive fracture in Th11–Th12, which was confirmed by the following computed tomography (CT) scan and magnetic resonance imaging (MRI) readings as well. Bone mineral density was 0.840 g/cm^2^ indicating osteopenia. Because of the family history, the patient underwent urological examination and ultrasound of the thyroid gland, liver, spleen and prostate. His lung CT scan was inconspicuous with small atelectasis in lower segments. He was also referred to a haematologist and endocrinologist, but after examination, both myeloma and parathyroid disease were excluded, although autoimmune thyroiditis due to raised autoantibodies against thyroid tissue was diagnosed. No treatment except for painkillers was prescribed.

**Table 1:** The sequence of laboratory findings from the beginning of disease

	2011		2012		2013	2014			2015
Sequence of analysis	1st	2nd	1st	2nd	1st	1st		2nd	(1 year after hip arthroplasty)
Laboratory findings									
Phosphate (0.87–1.45 mmol/l)	0.48					0.52	0.36		1.19
24 h urine sample phosphate (12.9–42.0 mmol/24 h)	26.49					17.97			%TPR = 90.52^a^
Calcium (2.15–2.50 mmol/l)	2.25		2.37			2.42	2.40		2.48
Ionised calcium (1.05–1.30 mmol/l)	1.12		1.16			1.30	1.09		
Alkaline phosphatase (40–150 U/l)	248	274	310	257.25	297.7	319.19	279		85.97
25-OH vitamin D (75–100 nmol/l)	78.4		54.68	47.93	77.70	85.83	72.3		44.42
Parathyroid hormone (1.6–7.2 pmol/l)			6.07	8.10		19.9	12.01		
FGF-23 26–110 U/l						589			104 (measured 3 months after hip arthroplasty)

^a^%TPR = 90.52—calculated according to the formula: 100 × (1 − ((urine phosphate/urine creatinine) × (serum creatinine/serum phosphate))). 100 × (1 − ((45.06/28.521) × (0.07/1.17))) and was found to be in normal range between 85 and 95 %. Numbers are entered in millimoles per liter

**Fig. 1:**
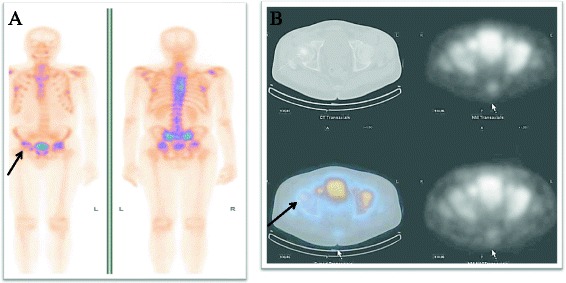
**a** Whole body bone scintigraphy 99mTc-MDP. Moderate uptake in the right shoulder, on both sides of the ribs due to osteoblastic lesions. Reduces radiotracer uptake in right femoral head (*long arrow*). **b** 99mTc-MDP SPECT/CT of the pelvis. Reduced radiotracer uptake in right femur head, corresponding sclerotic lesion on CT (*long arrow*) (year 2011)

With continuing weight and height loss together with bone and muscle pain, the patient was hospitalised at the Department of Rheumatology for thorough examination in November 2011. The alkaline phosphatase remained elevated and hypophosphatemia 0.48 mmol/l (normal values, 0.87–1.45) was documented for the first time. The 24-h urine sample phosphaturia showed normal phosphate clearance 26.5 mmol/24 h (normal values, 12.9–42.0 mmol/24 h) and subsequent phosphate readings in urine were within normal values as well. Vitamin D levels, calcium and ionised calcium readings were found within the normal values: vitamin D being 78.4 nmol/l (normal values, 75–100); calcium 2.25 mmol/l (normal values, 2.15–2.50); and ionised calcium 1.12 mmol/l (normal values, 1.05–1.30); nevertheless, he used periodically prescribed bone mineral supplements from the beginning of disease. The radiology of spine showed a new compressive fracture in the level Th7–Th8 in addition to the old fracture, which was later confirmed by CT scan. Whole body bone scintigraphy with 99mTc-MDP 550 MBq showed increased focal uptake of a radiotracer in the left shoulder and in the ribs on both sides due to osteoblastic process and reduced uptake in right femoral on SPEC/CT sclerotic bone lesion without tumour at that time or Morbus Paget specificity (Fig. 1). At that time, undifferentiated bone structural changes (M85.9) were diagnosed because of compressive fractures in spine, increased bone specific fraction of alkaline phosphatase and no evidence found for oncologic disease.

In February 2012, the avascular necrosis of both femur heads with osteosclerotic locus in the right femur of unknown origin was found by MRI. During routine follow-up in 2013, additional compressions were found in spine CT, now overtaking the segment from T5 to T12. The bone density continued to diminish up to 0.76 g/cm^2^ in the hip and spine, and alkaline phosphatase readings remained elevated. The antiosteoporotic treatment was started at that time with ac. zolendronicum (5 mg/100 ml) administered intravenously and repeated after a year of interval but with no clinical or laboratory improvement. It was discontinued and switched to oral bisphosphonate. Natrium risedronate was initially prescribed in dosage of 35 mg once per week and subsequently increased to 35 mg per day but was discontinued in the fall 2014 because of no effect.

**Fig. 2:**
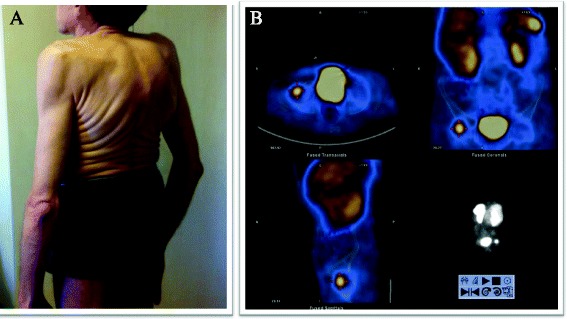
**a** The posture before the right hip endoprosthesis operation and tumour extirpation. The patient has given the written consent for his medical findings and photos to be published. **b** 99mTc-Tektrotyd whole body scintigraphy. Intensive, focal radiotracer uptake in right femur head (* red markers around*) (year 2014)

**Fig. 3:**
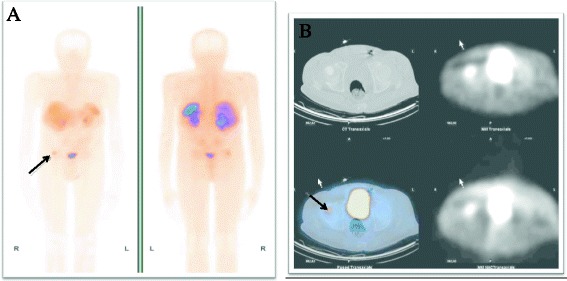
**a** 99mTc-Tektrotyd SPECT /CT of a pelvis. Focal, intensive radio tracer uptake in right femoral head (*long arrow*) and **b** corresponding sclerotic lesion on CT (*long arrow*) (year 2014)

**Fig. 4:**
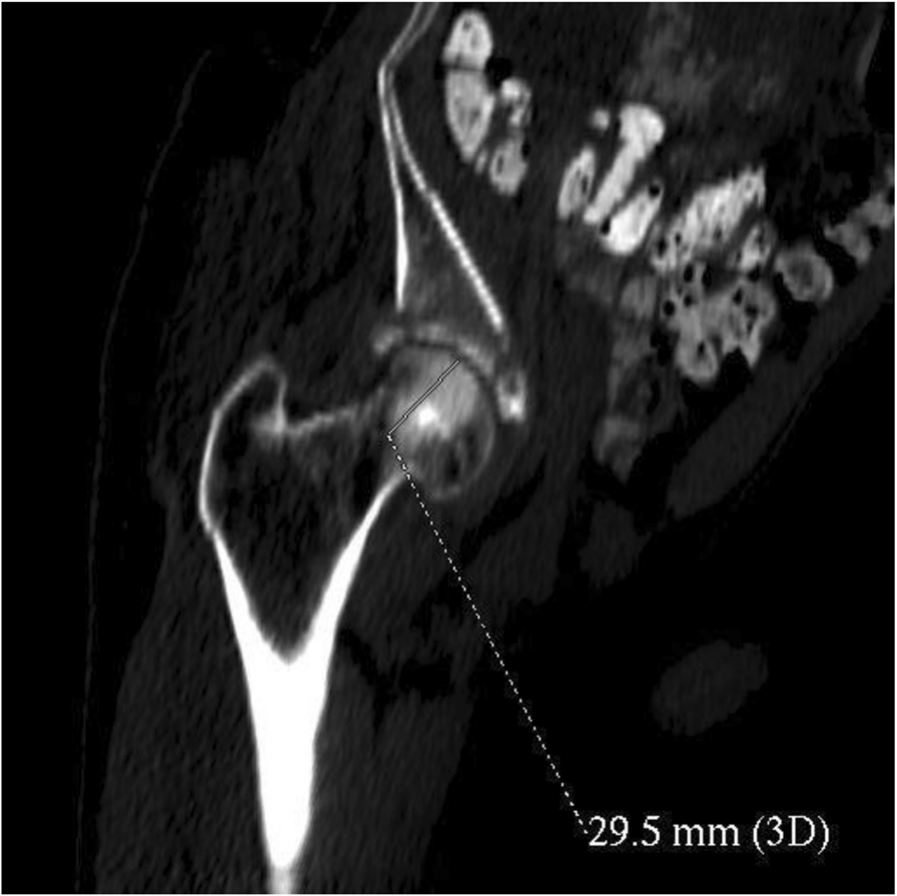
Osteosclerotic tumour in the right femur head revealed during the whole body computerised tomography (year 2014)

During the time period of almost 4 years, the clinical condition continued to worsen with a loss of height for 15 cm and weight for 20 kg from year 2011 now standing for 165 cm height and 53 kg weight (BMI = 23.1 kg/m^2^), permanent pain in chest and hips, progressing breathing discomfort, muscular weakness, inability to bend below knees and posture change (Fig. 2, photo taken in October 2014). Taking in account hypophosphatemia, elevated alkaline phosphatase from the beginning of disease, compressive fractures and avascular necrosis in femur heads, the differential diagnosis was broadened to acquired Fanconi’s syndrome, X-chromosome-linked hypophosphataemia and phosphaturic tumour-induced osteomalacia. To rule out Fanconi’s syndrome, the heavy metals analysis for copper, lead, cadmium and mercury were ordered and were found to be in normal ranges: copper 15.7 μmol/l (*n*, 11–22), lead 9.60 μg/l (*n*, 0–90), cadmium 0.8 μg/l (*n*, 0–1.7) and mercury <1.0 μg/l (*n*, 0–5). Because of disease manifestation in adulthood, no analysis for FGF gene mutation was requested. FGF-23 readings were found increased fivefold and being 589 U/l (normal values, 26–110 U/l). The whole body scintigraphy with somatostatin analogues 99mTc-tekrotyd 605 MBq revealed intensive uptake of radiotracer in the right femoral head, and SPECT/CT demonstrated uptake in sclerotic bone lesion in the right femoral head—tumour intensively expressing somatostatin receptors (Fig. 3). For possible multicentre tumour localization, the whole body CT was ordered. CT indicated deformations in skeletal bones, ribbons, thoracic vertebrae and pelvic bone due to osteomalacia or stress fractures, and no signs of healing were observed. CT also showed compressive fractures in Th3–Th11 with pathologic kyphosis, stress fractures in both femoral heads and osteosclerotic tumour in right femur head (Fig. 4). No deformations were found in soft tissues, parenchymal organs, lungs or lymph nodes.

**Fig. 5:**
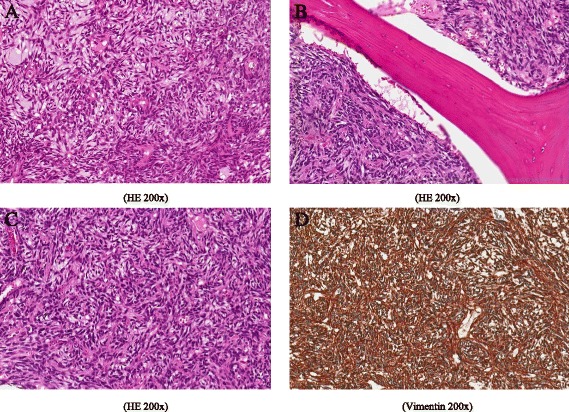
Histologic examination of the specimen revealed a hypercellular tumour composed of spindle-shaped cells in fibromyxoid matrix, with a hemangiopericytoma-like pattern (**a**). Tumour cells had clear to eosinophilic cytoplasm, nuclei were oval or elongated, of monomorphic appearance, with no signs of atypia. Mitotic figures were rarely seen. Surrounding bone trabeculae were irregular in size and shape with wide osteoid seams and foci of hyaline cartilage, showing enchondral ossification (**b**, **c**). The tumour cells were strongly and diffusely positive with vimentin (**d**) but exhibited no reactivity with PanCK, SMA, CD34, CD10 and D2-40 (year 2014)

With strong prediction of phosphaturic mesenchymal tumour-induced osteomalacia, a tumour-removal surgery was performed, and subsequently, removal of the head and the neck of the right femur was required following total hip arthroplasty in October 2014. Surgical specimen was presented by femur proximal head. Examination revealed ill-defined intramedullary grey tumour, approximately 2 cm in diameter. Bone tissue was soft, macroscopically intact by tumour and showed a broad band of cartilage under articular surface. Histologic examination of the specimen revealed a hypercellular tumour composed of spindle-shaped cells in fibromyxoid matrix, with a hemangiopericytoma-like pattern. Tumour cells had clear to eosinophilic cytoplasm; nuclei were oval or elongated, of monomorphic appearance and with no signs of atypia. Mitotic figures were rare. Surrounding bone trabeculae were irregular in size and shape with wide osteoid seams and foci of hyaline cartilage, showing enchondral ossification. The tumour cells were strongly and diffusely positive with vimentin but exhibited no reactivity with PanCK, SMA, CD34, CD10 and D2-40 (Fig. 5).

**Fig. 6:**
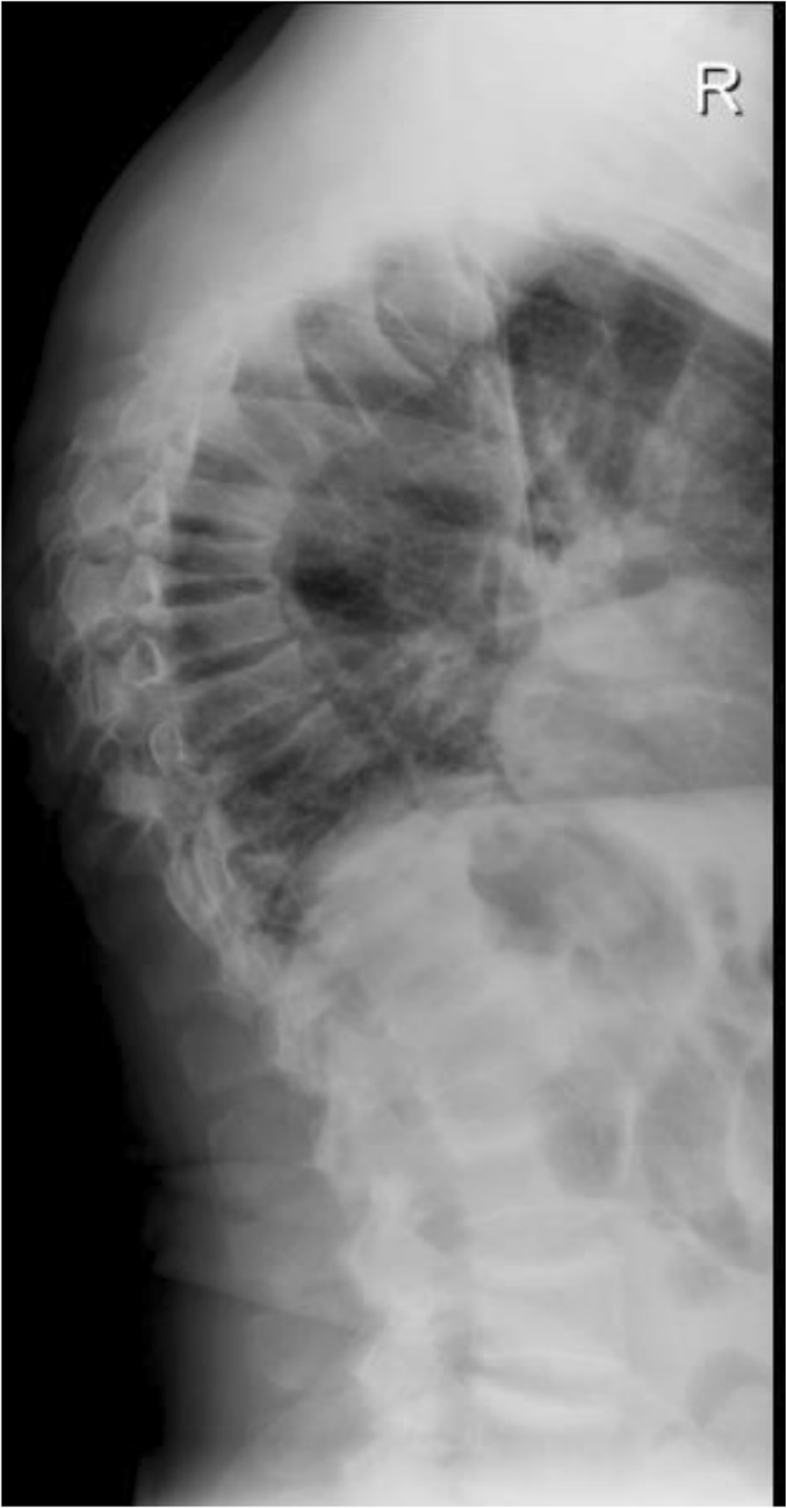
Conventional radiography of the spine after 1 year after tumour resection showed permanent and nonreversible spine deformation due to the old compressions from Th3 to Th11 with no evidence of new compression fractures (year 2015)

The post operation period went without clinical complications and after 3 months the levels of phosphorus, alkaline phosphatase and FGF-23 were measured. Alkaline phosphatase remained elevated 316 IU/l (40–150) with bone fraction now standing at 57.7 % and FGF-23 returned to normal values 104 IU/l (26–110 IU/l). Three months after surgical procedure, the body pain almost disappeared and the weight increased up to 62 kg and height up to 166 cm (BMI = 22.5 kg/m^2^). The alkaline phosphatase returned to normal values 6 months after tumour extirpation and remained within normal values after 1 year of follow-up (Table 1). The phosphorus level increased remarkably up to 1.51 mmol/l (normal range, 0.87–1.45) after 3 months and normalised within 1 year. We have to admit that measuring 24-h urinary phosphate was not a proper phosphate balance measure throughout the disease course. Without applying more sophisticated methods, e.g. percent tubular reabsorption of phosphate (%TRP) or tubular maximum for phosphate corrected for glomerular filtration rate (TmP/GFR) as it is recommended [16], significant loss of phosphate was probably missed from the beginning of disease. Conventional radiography of the spine after 1 year showed permanent and nonreversible spine deformation due to the old compressions from Th3 to Th11 with no evidence of new compression fractures (Fig. 6).

## Conclusions

The diagnosis of TIO is a challenge under the best of circumstances. TIO should be included in the differential diagnosis in patients with progressive weakness, bone and muscle pain and multiple fractures. The diagnosis is commonly delayed for years due to the nonspecific nature of the presenting symptoms, failure to include determination of serum phosphorus levels in blood chemistry testing and difficulty in identifying the responsible tumour. The professional evaluation of whole body images, including single-photon emission tomography enables identification and better tumour contrast which can be found in skeletal structures of whatever region of the human body. FGF23 may be of help to understand the underlying mechanisms of TIO and a measurement to follow up the disease course.

### Ethics statement

This review includes the description of one case report, and the consent form for publication was obtained from the person. The consent form of that particular person was sent to the Editor of this journal.
